# Using economic evaluations in implementation science to increase transparency in costs and outcomes for organizational decision-makers

**DOI:** 10.1186/s43058-022-00295-1

**Published:** 2022-04-11

**Authors:** Lisa Saldana, Debra P. Ritzwoller, Mark Campbell, Eryn Piper Block

**Affiliations:** 1 Oregon Social Learning Center, Eugene, OR USA; 2 Institute for Health Research, Kaiser Permanente Colorado, Denver, USA

**Keywords:** Implementation cost, Resources, Decision-makers, Economic evaluation, COINS, Cost-effectiveness, Stages of Implementation Completion

## Abstract

**Background:**

Economic evaluations frequently are utilized to compare the value of different interventions in medicine and health in concrete terms. Implementation science also would benefit from the incorporation of economic evaluations, but such studies are rare in the literature. The National Cancer Institute has supported a special collection of articles focusing on economic evaluations in implementation science. Even when interventions are supported by substantial evidence, they are implemented infrequently in the field. Implementation costs are important determinants for whether organizational decision-makers choose to adopt an intervention and whether the implementation process is successful. Economic evaluations, such as cost-effectiveness analyses, can help organizational decision-makers choose between implementation approaches for evidence-based interventions by accounting for costs and succinctly presenting cost/benefit tradeoffs.

**Main text:**

This manuscript presents a discussion of important considerations for incorporating economic evaluations into implementation science. First, the distinction between intervention and implementation costs is presented, along with an explanation of why the comprehensive representation of implementation costs is elusive. Then, the manuscript describes how economic evaluations in implementation science may differ from those in medicine and health intervention studies, especially in terms of determining the perspectives and outcomes of interest. Finally, referencing a scale-up trial of an evidence-based behavioral health intervention, concrete case examples of how cost data can be collected and used in economic evaluations targeting implementation, rather than clinical outcomes, are described.

**Conclusions:**

By gaining a greater understanding of the costs and economic impact associated with different implementation approaches, organizational decision-makers will have better transparency for future replication and scale-up. The use of economic evaluations can help to advance this understanding and provide researchers, purveyors or third-party intermediaries, and organizational decision-makers with essential information to facilitate implementation.

**Supplementary Information:**

The online version contains supplementary material available at 10.1186/s43058-022-00295-1.

Contributions to the literature

- Outlines the critical need for economic evaluations to inform researchers, purveyors, and decision-makers about the most cost-effective implementation strategies to use for their resource context.
- Defines the difference between evaluating the resources and costs of the intervention versus the full implementation process.
- Argues for perspectives and outcomes to be catered to the different priorities and goals of implementation science versus traditional health economics.
- Highlights pragmatic implementation models and cost-mapping tools that can be employed in real-world settings.
- Uses a case study to illuminate why it is important to have accurate implementation cost estimates and how economic evaluations can be incorporated into implementation studies in practice, featuring a well-established cost-mapping tool.

## Background

Economic evaluations, such as cost-effectiveness analyses, are frequently utilized in medicine and health to compare the value of different interventions in concrete terms. Such economic evaluations also would be useful for understanding the comparative value of different implementation methods in implementation research, but are rare in the literature [1]. Implementation science exists, in part, because even when an intervention is well-studied, and has been found to be highly effective and cost-effective, most organizations that consider implementing it will fail to bring the intervention to fruition. In child public service systems, it is estimated that over 90% of agencies fail to implement evidence-based practices [2, 3].

Studies show that financial and resource costs during the implementation process are substantial determinants of the likelihood of the adoption and sustainment of evidence-based practices [4–7]. These are costs over and above the direct costs of the intervention. Indeed, one qualitative evaluation of decision-makers operating in a range of service settings found that the costs of pre-implementation activities, including changing workflow, modifying contracts, and building infrastructure, were predominant factors in the decision of whether or not to implement various evidence-based practices [8]. Implementation costs often are overlooked in economic evaluations [9], and there are few economic evaluations that compare the value of implementation strategies, incorporating such implementation costs [10, 11].

### Contribution to the literature

In this paper, we argue that (1) transparency in implementation costs is necessary for informed decision-making and (2) economic evaluations are useful tools for implementation research, but in many cases, they must be catered to the needs of organizational decision-makers, prioritizing pragmatism over perfection. Using a case study, we present novel strategies for incorporating economic evaluations into implementation research, specifically to compare different implementation approaches for the same intervention. First, we will describe the differences between intervention and implementation costs. Then, we will define how economic evaluations in implementation science may differ from those in medicine and health intervention studies, especially in terms of determining the perspectives and outcomes of interest. Finally, given the limited examples from the field, we will use a case study to illuminate why it is important to have accurate implementation cost estimates and how economic evaluations can be incorporated into implementation studies in practice. The case study features a costing tool, the Costs of Implementing New Strategies (COINS), which can be used in a range of implementation-based economic evaluations.

## Main text

### Implementation versus intervention costs

There is an important, but often overlooked distinction between costs related directly to an intervention, and those related to the full implementation process for said intervention. For evidence-based practices, the resources and costs specific to the intervention itself often are explicit, having been defined through previous randomized trials when building the original evidence base. In the case of evidence-based interventions, estimates of the direct costs associated with the intervention are often straightforward to capture and quantify. For example, costs for training, materials, or technology might not differ by setting or target population. Similarly, contact, supervision, and other intervention costs that increase or decrease as a function of the number of entities targeted may be consistently captured via staffing logs or other standard cost-capture instruments [12, 13].

Yet, even with a well-established evidence-based program, costs related to the *implementation* of the intervention are difficult to define and estimates of overall implementation costs are rare. Implementation costs are incurred while building the infrastructure needed to support the program development, engaging stakeholders, delivering the intervention, and sustaining the intervention. Even the more commonly used strategies (e.g., stakeholder meetings, coaching, tailoring) differ in required staff resources and costs depending on the complexity of the intervention and/or the multi-level (e.g., community stakeholders, providers, patients) and multi-component nature of the implementation strategies selected (see Eisman et al. [5]).

Intervention costs often are a mixture of fixed, invariant, and variable costs, whereby the variable inputs are a function of the size of the target population [14]. While the costs of the implementation also are fixed and variable, they are dependent on the implementation strategy selected and the quality of implementation activity completion. To properly plan out the implementation of a new program, decision-makers need to know the financial costs, but also how much staffing time, building space, and other non-financial and indirect resources will be used. Many of these non-financial resources are unforeseen in the implementation process, even when many resources already are accounted for in the costs of the intervention itself. Cost estimates of *both* the intervention and implementation must be available in order for decision-makers to have realistic expectations regarding the feasibility of fully implementing new programs. Cost-mapping tools for documenting and organizing such costs are discussed below.

### Considerations for incorporating economic evaluations into implementation studies

Most economic evaluations in health and medicine are focused on interventions themselves rather than the implementation of interventions, and the standards for these evaluations presented by the field of health economics reflect this distinction. The panel on cost-effectiveness in medicine and health recommends that all health-focused economic evaluations incorporate two reference cases: one for the health system perspective and one for the societal perspective, and only in some cases includes a third perspective of a specific payer or institution. In economic evaluations, a *perspective* is the “viewpoint from which a cost-effectiveness analysis is conducted” [15]. Having the system and societal reference cases in economic evaluations of an intervention—as opposed to an implementation approach—is important because (1) they improve comparability of evaluations, (2) they decrease the likelihood that costs are arbitrarily externalized or the long-term costs or benefits for individuals are overlooked, and (3) they encourage researchers and decision-makers to think about health in its broadest sense rather than a series of costs and financial gains for a specific payer [16].

On the contrary, a narrow perspective often is more useful than the broad health system or societal perspective for implementation studies. Specifically, the perspective of the organizational decision-maker (e.g., clinic directors, system leaders, program managers) is often the priority [17]. Implementation science is a pragmatic science; implementation trials have an implicit goal of generating pragmatic information that will bridge the gap between research and practice for programs that already have a strong empirical evidence base. Thus, the goals of an economic evaluation in implementation science are more focused on application. Implementation trials often make the assumption that the implementation approach does not explicitly impact the quality of the intervention outcomes, as long as implementation is successful.

Implementation-focused economic evaluations also are likely to have different outcomes of interest than intervention-specific evaluations. For interventions, many outcomes are at the individual level, such as quality-adjusted life years, in order to generally quantify the intervention’s health effect [18]. Conversely, implementation studies often focus on organizational outcomes such as adoption, feasibility, fidelity, penetration, reach, and sustainability [19]. This might also mean that the time horizon is shorter since long-term patient outcomes are outside of the scope of these studies.

### Recommended implementation science components for economic evaluations

This section presents how an implementation process framework, a model, and a cost-mapping tool can be integrated for the purposes of economic evaluations. There are over 150 implementation theories, models, and frameworks currently [20], but we focus on the Exploration, Preparation, Implementation, and Sustainment (EPIS) [6] framework, the Stages of Implementation Completion (SIC) process model, and the Cost of Implementing New Strategies (COINS) cost-mapping tool. The EPIS framework describes the phases of the implementation process from the point of exploring which intervention is most appropriate to meet identified needs, through the point of sustaining it [21]. Throughout all four defined phases of implementation, the role of funding is delineated as a critical factor to consider, particularly in the outer context. EPIS can be seen as an overarching framework that informs and helps synthesize other implementation tasks/tools [21].

The Stages of Implementation Completion (SIC) model stages [22] align with the phases of the EPIS framework. SIC is an 8-stage tool for assessing and monitoring the implementation process, moving from pre-implementation, to active implementation, and to sustainment (Table 1). Each SIC stage is populated with a range of implementation activities, including those that are applied broadly across interventions (e.g., training provider staff) and those specific to an intervention (e.g., recruitment of foster parents [22]).

**Table 1:** The Stages of Implementation Completion (SIC) with example items for each stage

Stage	Stage	Example Item
*Pre-Implementation Phase*		
*Stage 1*	Engagement	Date organization agreed to implement program.
*Stage 2*	Consideration of Feasibility	Date of stakeholder feasibility meeting
*Stage 3*	Readiness Planning	Date of cost calculator/funding plan review
*Implementation Phase*		
*Stage 4*	Staff Hired and Intro Training	Date hired program supervisor.
*Stage 5*	Fidelity Monitoring Processes in Place	Date tested audio recording equipment.
*Stage 6*	Services and Consultation to Services Begin	Date first client received intake assessment.
*Stage 7*	Model Fidelity and Staff Competence and Adherence Tracked	Date 50% of provider staff achieved passing fidelity.
*Sustainment Phase*		
*Stage 8*	Competency	Date program graduated tenth client successfully.

Cost-mapping tools serve as an important companion to the process of operationalizing an implementation approach in order to document relevant costs and disentangle implementation from intervention costs. It is important to identify the perspective of the analysis to determine which costs to capture. In this case, we focus on the costs accrued by a specific payer rather than the broader costs to society. The Cost of Implementing New Strategies (COINS) is one such cost-mapping tool, which was developed specifically as a standardized approach for mapping costs associated with implementation activities defined on the SIC [23]. COINS helps capture the full spectrum of identified costs and unaccounted for personnel effort necessary to build the infrastructure and support for successful implementation and sustainment. It was used in the case study below.

The COINS tool is similar to economic approaches such as time-driven activity-based costing (TDABC) [24]. When used to cost implementation, this method includes identifying each component resource unit, assigning their value, and aggregating across the intervention and implementation components. TDABC encourages the assessment of time and costs needed to conduct clearly defined implementation strategies, providing a method for increasing transparency with decision-makers regarding the time and associated costs to expect for completing the implementation.

### Case study

To demonstrate the integration of implementation science and economic evaluations across the phases of implementation, this case study presents a large randomized implementation trial comparing two implementation strategies for the same evidence-based practice (PI: Chamberlain [25]).

#### Summary of trial

The original trial examined two approaches for implementing Treatment Foster Care Oregon (TFCO; formerly known as Multidimensional Treatment Foster Care), an intervention developed as an alternative to residential placement or congregate care for youth with severe behavioral and mental health problems within sites in California and Ohio at the county level [26]. While engaged in TFCO, youths are placed with specialized foster parents who have been intensively trained and receive consistent supervision, support, and mentoring. Youths typically stay with their TFCO foster parents for 6 to 9 months and engage in activities tailored to their individual needs such as strength-based behavior management training, individual and family therapy, social skill training, and case management [27]. TFCO is backed by multiple randomized controlled trials and has been found to reduce the likelihood of adverse outcomes such as youth recidivism, delinquency, deviant peer relations, internalizing symptoms, psychotic symptoms, and unplanned pregnancy [28–32].

The two implementation approaches examined to implement TFCO included (1) standard individual (IND) purveyor support or (2) Community Development Teams (CDT)—a manualized learning collaborative, with organizations from six regionally associated counties teamed together, facilitated by two experts [33]. The IND purveyor support model follows the traditional implementation support process of 1-1 consultation between an evidence-based practice expert purveyor (most often affiliated with the developer) and an adopting organization. Through a series of calls and site visits, the TFCO purveyor guides the organization through the implementation process, offering support and review along the way. On the other hand, the CDT collaborative model utilizes less frequent but more intensive daylong meetings with a cohort of organizations all focused on implementing the same intervention [34]. With the assistance of a CDT facilitator, CDT engages adopters in peer-to-peer networks to work through implementation struggles together, share solutions, and develop best practices [33]. This trial tested the hypothesis that the cohort-driven CDT approach was more likely to lead to the successful implementation of TFCO than standard individual methods (IND) in which the adopter worked directly and solely with the intervention purveyor [35]. Counties were randomized to one of the two implementation strategies to support the completion of feasibility and readiness activities, as well as other non-clinical implementation supports [25]. Counties selected a provider organization to implement the TFCO intervention. Both conditions received the same level of clinical consultation and technical support for intervention delivery.

#### Assessment of implementation costs

**Fig. 1:**
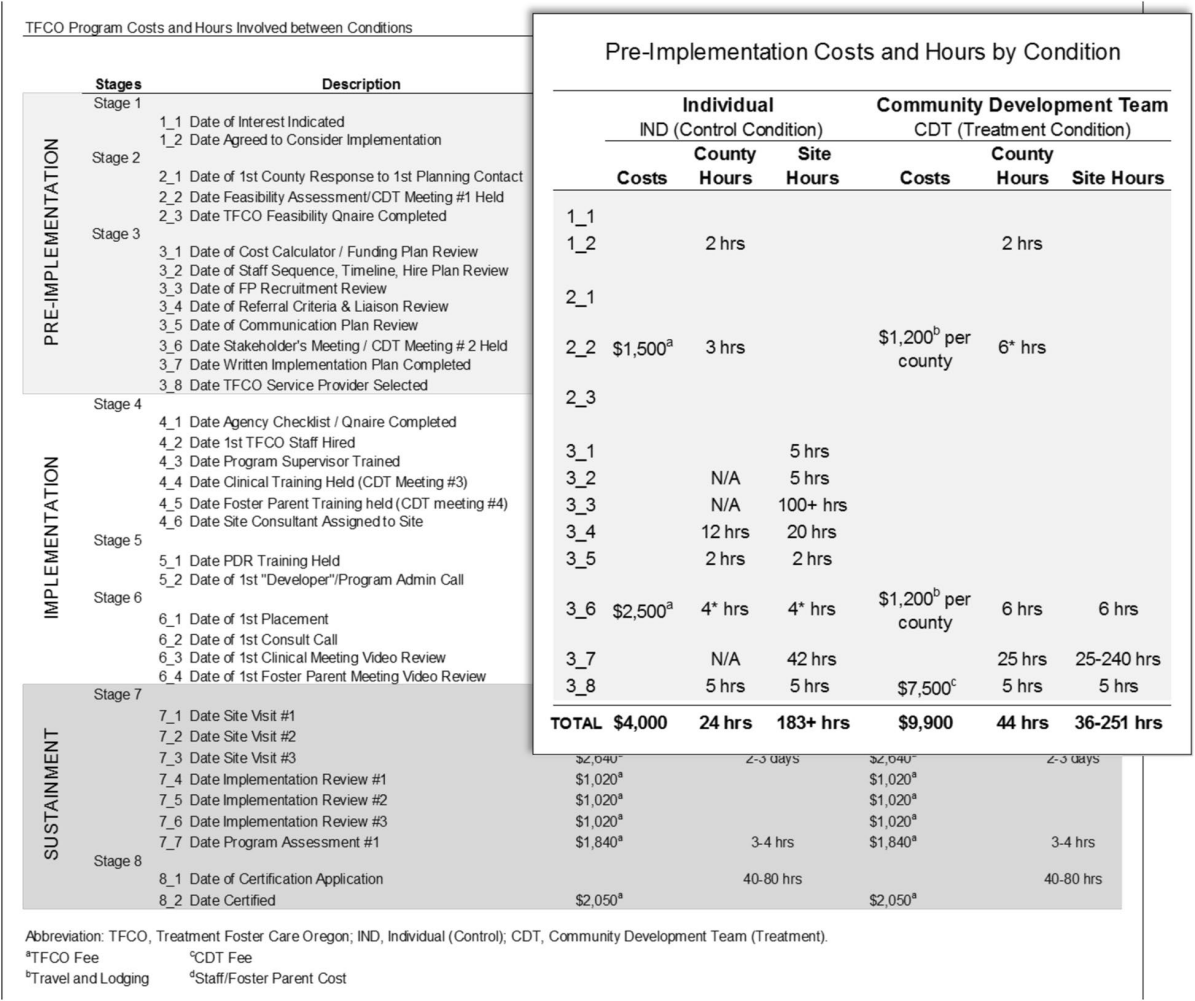
Example of data collected using the COINS tool. Legend: Original full data reported in Saldana et al., 2014

The previously described COINS cost-mapping method was developed as part of this trial and was used to measure variation in resource and cost needs for implementing TFCO using the IND versus CDT implementation approach [23]. Figure 1 provides a condensed illustration of outcomes produced by this approach. To help capture this data, the COINS tool is an interactive, online portal that is integrated with the SIC implementation process data collection and tracking web-based platform. As organizations complete each implementation activity defined on the SIC (e.g., external stakeholder meeting), data is entered about the time of completion, number and type of staff hours used, and various financial costs associated with completing this implementation activity [36]. Because data is collected prospectively as the implementation progresses, the COINS decreases the data collection burden over retrospective recall or collection. The online tool can then summarize cost information as in Fig. 1 for analysis.

#### Possible uses of economic evaluations in this case study

Economic evaluations are useful tools for making assumptions, priorities, risks, and trade-offs explicit for complex situations [17] and include such methods as cost-benefit, cost-effectiveness, cost-utility, and budget impact analysis (an explanation of each of these can be found in Table 2). Data collected using the COINS method from organizations in 51 counties across each of the implementation phases provided the opportunity to conduct such economic evaluations. This section is structured by the implementation phase and the SIC stages that map onto them. For each implementation phase, outcomes of interest and appropriate economic evaluation types are presented and discussed.

**Table 2:** Economic Evaluation Types with Descriptions and Examples from the TFCO Case Study

Economic Evaluation Type	Description	Outcome type	Example Perspective	Example Description (using TFCO)
**Return-On-Investment Analysis**	A direct analysis of the level of returns projected for a specific investment, relative to the cost of that investment	Ratio of gain/loss relative to cost	Narrow – organizational decision-maker	To determine the likelihood of sustainment, calculate the estimated costs for full implementation and project the number of patients needed to recoup those costs
**Budget Impact Analysis**	Narrow analysis of the impact of a program on the budget of a specific payer. Does not take into account the broader costs or benefits to a larger social system or to society	Financial impact on a specific payer	Narrow – organizational decision-maker	An organization that is interested in implementing TFCO can project the specific costs related to implementation and the intervention itself using previous estimates collected with COINS, then calculate the projected financial returns from Medicaid and foster care placement billing
**Cost-benefit**	All costs and benefits translated into financial terms. Could include costs and benefits at the societal or broader system scale. However, not often used in health or social service research because it can be challenging (and controversial) to translate human life outcomes into monetary terms	All outcomes measured in monetary terms	Broad – Systems perspective (social services)	Although the implementing organization may recoup costs directly through billing, other cost-savings may occur across the social service systems. A cost-benefit analysis could estimate the cost savings related to lower levels/intensity of crime, recidivism, and behavioral health treatment for the patients of TFCO.
**Cost-effectiveness**	Estimating the costs spent to increase one unit of health or social outcome. Usually considers the broader society or health/social service system rather than just one payer	Units of health or social outcome such as years of life saved or fidelity score	Narrow – organizational decision-maker	For IND and CDT, there are risk probabilities of implementation failure at each step of the SIC process which map onto different likely costs. If an organizational decision-maker is only willing to risk a certain amount of sunk costs, cost-effectiveness analysis using a decision-tree could help them understand which implementation strategy (IND or CDT) is less likely to exceed that threshold (see Appendix A for decision tree example). In implementation science, relevant health outcomes include adoption, fidelity scores, patient reach, penetration, and sustainment
**Cost-utility**	Similar to cost-effectiveness, but a more specific analysis that summarizes health outcomes into both mortality and morbidity.	Units of health or social outcome accounting for quality of life (such as Quality Adjusted Life Years (QALYs))	Broad - Systems and Societal	An evaluation of both the implementation approaches (CDT vs. IND) and the intervention, TFCO, itself would include both implementation outcomes such as reach and penetration as well as patient outcomes such as youth recidivism, delinquency, internalizing symptoms, deviant peer relations, psychological symptoms, and unplanned pregnancies

In this case study, we make the assumption that the patient outcomes of the intervention itself would be equivalent regardless of the implementation approach, were the intervention to be implemented with competence. Thus, we will not focus on implementation-based outcomes that would impact intervention quality such as fidelity. To support our goal of highlighting ways in which economic comparisons can inform transparent decision-making, we focus on the perspective of the organizational decision-maker (e.g., clinic directors, system leaders, program managers) rather than the larger societal or systems perspective.

##### Pre-implementation (SIC stages 1–3)

When determining whether to implement a program, organizational decision-makers need information about the likelihood that they will be able to successfully implement that program and the costs (financial and otherwise) they are likely to expend throughout the process. In the current case study, the two implementation approaches incurred different costs at each of the three stages of pre-implementation, resulting from their inherent structures and business models. The standard individual (IND) purveyor approach asked organizations to sign a contract with pre-payment of fees associated with completion of the readiness process (prior to SIC Stage 3), while the Community Development Team (CDT) requested payment for implementation support after the readiness stage was completed. Although CDT sites accrued costs in time and travel for in-person group meetings during the readiness process, there were minimal additional direct costs until readiness was complete.

The variation in the timing of these costs between conditions during pre-implementation had significant implications for potential sunk costs to the decision-maker, particularly for programs that ended up not being successful. Although organizations did not show substantial differences in the rates of successful progression through SIC stage 3 readiness (40% in CDT; 36% in IND), those from the IND condition that discontinued had substantially greater sunk costs toward unsuccessful programs.

In this case—which includes multiple stages, with different costs and different probabilities of success at each stage—a decision tree would be a useful tool to model cost-effectiveness for the perspective of the organizational decision-maker [37]. By inputting the likely costs, outcomes, and probabilities of success into a visual, branching model, decision-makers would be better able to understand the trade-offs between the timing of costs and the likelihood that those costs will lead to the adoption of the program (see Appendix A for an example decision tree).

##### Implementation (SIC stages 4–7)

During the implementation phase, organizations incur costs related to hiring or assigning staff to the new program, training them, overhead expenses for office space for the new hires, purchasing equipment for them to perform their tasks, and initiating the referral flow process to begin screening youth for services. While the costs associated with hiring, training, and direct expenses such as equipment were approximately the same across conditions, the level of support, and interaction received by either the IND purveyor or the CDT group differed, with those in the CDT condition working collectively through policy, implementation, and referral challenges whereas those in the IND condition worked independently and directly with the purveyor. (See Saldana et al., (2014) for an in-depth explanation of the differences in time and effort spent between conditions [23]).

One important outcome of the implementation phase is the penetration rate—the number of organizations who start a program compared to the total number of organizations who agreed to begin the pre-implementation phase. In both implementation research and real-world scale-up efforts, the rate of penetration is particularly useful when considering cohort outcomes. For system leader decision-makers, this metric can inform the likelihood that scale-ups across networks will successfully penetrate the system. The unit of analysis is the organization. For the current case study, a cost-effectiveness analysis from the perspective of the organizational decision-maker includes costs per condition divided by the number of organizations starting a program to determine the average cost of program launch per condition. Both the sunk costs for discontinued sites in that condition and the start-up costs for successfully launched sites would be included. As an example, at $7,277,618, the total costs (as recorded with the COINS tool) across all 10 CDT organizations that started a program far exceeded the $3,342,070 incurred by the 9 organizations implementing IND.

A second important outcome during the implementation phase is reached–the number of youth served per organization: the unit of analysis is the individual youth

Once the first youth is served, the organization begins to receive financial returns by billing Medicaid for behavioral health therapy and from the state for youth foster care placement. Yet, organizations also continued to incur ongoing implementation costs to operate the program and to work toward the development of competency in program delivery. A cost-effectiveness analysis could illuminate the relationship between patient reach, ongoing costs, and financial returns. The above example of penetration rates suggests that program launch for CDT organizations was more costly than that of IND organizations. However, because of the significantly greater number of youth served (i.e., patient reach) by CDT organizations (152 youth in CDT compared to 59 in IND), the average cost per youth was lower for CDT organizations ($47,879 for CDT compared to $56,645 for IND). Therefore, organizations implementing the CDT implementation strategy both served more youth and did so at a lower average youth cost than those implementing IND.

##### Sustainment (SIC stage 8)

Finally, from an organizational decision-maker’s perspective, there is a need to understand when and how costs would be recouped from implementing a new program in order to develop a plan for financial sustainment. This could include a return-on-investment analysis, calculating the point at which the financial gains surpass the cost estimates based on the number of youth served. In this case study, sites varied greatly in their staffing costs, relating to the timing of hiring and FTE levels rather than salary differences—some sites hired staff far before any youth were served and thus paid those staff during times they were not yet working directly with youth. The study period was the time horizon used for this analysis. Of those that sustained through the study period, on average, organizations that placed more than 10 youth maintained a positive gain from reimbursement above program implementation and delivery costs during the time horizon of the study. Organizations that gained approximately $140,000 on average recovered their start-up costs.

This analysis does not measure long-term financial sustainability (i.e., the number of youth necessary to be consistently served in order to continue to break even). In order to conduct such an analysis, a per-youth-per-month rate would be necessary. Additionally, higher-level decision makers may also be interested in an analysis of the average cost-effectiveness ratio at the end of the study period of all organizations who began the pre-implementation process, whether or not they reached the sustainment phase, incorporating sunk costs incurred by organizations that discontinued the program. However, that is beyond the scope of this specific analysis, which focuses on the narrower organizational perspective.

## Discussion

This manuscript argues that economic evaluations are useful tools for implementation studies, but in many cases, the organizational decision-maker perspective is more salient than the societal or system perspective. Economic evaluations are useful because they make costs, assumptions, risks, and benefits explicit where they often are implicit or hidden.

The case study presents a set of examples of the uses of economic evaluations for implementation science and integrates practical tools, specifically COINS and SIC into the measurement approach. In comparing potential implementation strategies (CDT vs IND), incorporating costs and resources illuminates some of the nuances that differentiate the utility of the two strategies for organizational decision-makers. The case study operates under the assumption that the effects of the intervention would be equivalent regardless of the implementation approach, so long as the patient receives the intervention. Under these conditions, the organizational decision-maker is an appropriate perspective to take for an economic evaluation in implementation science.

There are many instances in which societal and systems-level perspectives should be included in implementation-based economic evaluations, for instance, if the study evaluates the effectiveness of the intervention itself in relation to the implementation methods. This would be especially helpful in cases in which the implementation strategies differ not only in the penetration and reach but also in the quality of intervention outcomes at the patient level (for instance, in the case of TFCO, variable rates of youth delinquency, or internalized symptoms) (see Table 2). Additionally, prospective cost-mapping tools like COINS could help disentangle implementation costs and benefits at multiple levels, such as the organization, the system, and the state, and taking a broader perspective could illuminate ways in which a state could benefit from interventions that seem to be costly upfront but have long-term benefits to entities under the state’s purview.

Yet, it is important to remember that the priorities and goals of implementation science often are different from intervention-specific evaluations and should be treated as such. Since implementation science aims to bring programs that have already been proven effective into practice on a broader scale, studies in implementation science ask different questions, such as the following:

          How do implementation strategies differ in:

- The probability of failure at each stage?
- The costs of each stage?
- The types of costs (direct versus personnel effort) in each stage?
- The average penetration rate and its associated costs?
- The average number of patients served and the cost per patient?
- The number of patients needed to be served in order to recoup upfront implementation costs and become financially sustainable?

Regardless of the questions being answered, the implementation processes needed for public health efforts often require the coordination of multiple individuals, systems, and suppliers, with the resulting needed resources for quality implementation posing barriers for many communities. Without transparency in these resource needs, communities are not provided with realistic expectations for implementation and ultimately risk using what few resources they have available on a program that is not set up for success. This is especially true for communities with limited resources. Equipped with knowledge about the full costs at each step of implementation, decision-makers can decide where to invest their resources.

### Recommendations and next steps

This overview and case example suggest several recommendations for future research. There is a need for greater understanding and transparency of the costs associated with implementing evidence-based programs. Methods such as COINS, TDABC, or other micro-costing approaches provide pragmatic approaches for disentangling implementation from intervention costs and help to move from theoretical discussion of the importance of cost and resource considerations to application of economic evaluations to inform practice.

In order for economic evaluations to be useful in implementation science, the perspective and outcomes often differ from those in health science or intervention-specific studies. Since implementation science has pragmatic aims, the perspective of the organizational decision-maker often is prioritized and process and organizational outcomes are the main focus. These decisions, and the assumptions that come with them, should be made explicit in each study.

Although every implementation is unique in its setting and context, there is benefit in understanding case examples, especially when those examples are conducted under rigorous conditions with a comparator condition. Beyond contributing to the literature, case examples can inform real-world practice. For instance, the TFCO case example informed changes to the implementation strategy of the TFCO purveyor organization. Since the end of the study period, the purveyor organization has been operating under an improved business model more aligned with a phasic approach to implementation and considers the use of organizational cohorts when appropriate, having learned from the outcomes presented here.

## Conclusions

There is a growing interest in the use of economic evaluations in implementation science as evidenced by the special collection of articles supported by the National Cancer Institute (2021). Traditional methods used by health economists to evaluate the effectiveness and benefit of clinical outcomes also are relevant for implementation questions. Although the targeted outcomes might be at the organization or community level rather than the level of the individual patient, basic methods for costing and evaluating costs often can be applied. For decision-makers, knowing the estimated costs for the intervention is necessary, but not sufficient, for having reasonable expectations for resources needed to adopt a new intervention. We have the basic tools and methods to improve understanding of the costs and economic impact associated with different implementation strategies, thereby increasing transparency and efficiency. In so doing, we improve our potential to provide the confidence to organizational decision-makers to consider adopting evidence-based practices and policies.

## Supplementary Information

**Additional file 1.** Example decision tree for a cost-effectiveness analysis of pre-implementation comparing CDT and IND implementation approaches.

## Data Availability

Data used for the case examples were from a previously reported trial. Data might be obtained upon request from the original study principal investigator, PattiC@oslc.org.

## Abbreviations

QALYs: Quality-adjusted life years saved
EPIS: Exploration, Preparation, Implementation, and Sustainment
TDABC: Time-driven activity-based costing
COINS: Cost of Implementing New Strategies
SIC: Stages of Implementation Completion
TFCO: Treatment Foster Care Oregon
IND: Standard Individual Purveyor Support
CDT: Community Development Teams
