# Supplementary material for: Using economic evaluations in implementation science to increase transparency in costs and outcomes for organizational decision-makers
Source: Implement Sci Commun. 2022 Apr 11;3:40. doi: 10.1186/s43058-022-00295-1 (PMC9004101; doi:10.1186/s43058-022-00295-1)
Supplement: Supplementary file 1 — Additional file 1. Example decision tree for a cost-effectiveness analysis of pre-implementation comparing CDT and IND implementation approaches. [file 43058_2022_295_MOESM1_ESM.docx]

**Appendix A: Example Decision Tree for a Cost-Effectiveness Analysis of Pre-Implementation Comparing CDT and IND Implementation Approaches**


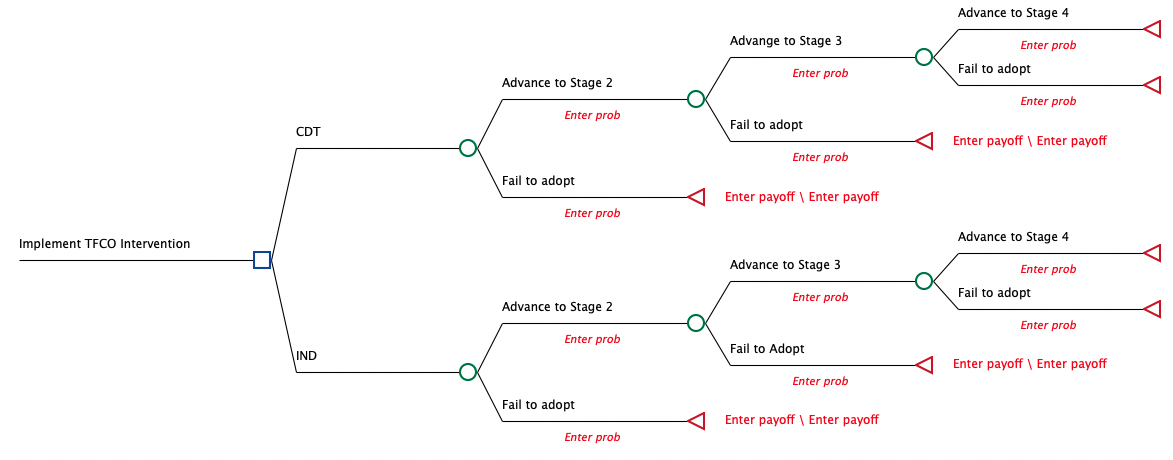


**Notes:** The blue box denotes the decision node at which point the organizational decision-maker would choose between the two implementation approaches. The green circles are chance nodes, and each branch after a chance node is assigned a probability (with all branches directly stemming from a chance node summing to 100). The red triangles are terminal nodes and are assigned a pay-off or cost. By calculating the collective probabilities of failure and the costs, one can compare the relative cost-effectiveness of each implementation approach. This diagram was created in TreeAge Pro Healthcare 2022 Software.
